# The evolutionary functions of consciousness

**DOI:** 10.1098/rstb.2024.0299

**Published:** 2025-11-13

**Authors:** W. Tecumseh Fitch, Colin Allen, Adina L Roskies

**Affiliations:** ^1^ Department of Behavioral and Cognitive Biology, University of Vienna, Vienna, Austria; ^2^ Department of Philosophy/Department of Psychological and Brain Sciences, University of California Santa Barbara, Santa Barbara, CA, USA

**Keywords:** consciousness, evolution of cognition

## Abstract

*Why* did consciousness evolve? Assuming that some species (e.g. humans) have consciousness and others (e.g. redwoods or mushrooms) do not, what problem(s) did consciousness evolve to solve? From a biological and evolutionary viewpoint, and regardless of which species have consciousness (or to what degree), this question of the adaptive function(s) of consciousness is central. Nonetheless, the growing discipline of consciousness studies has not yet fully engaged with this issue. The current special issue aims to help fill this important gap in the literature with contributions from 28 noted scholars in the field. In this introduction, we discuss basic terminological issues and potential pitfalls, provide a broad theoretical framework, consider some of the many possible answers to this central question and offer brief summaries of the included papers.

This article is part of the theme issue ‘Evolutionary functions of consciousness’.


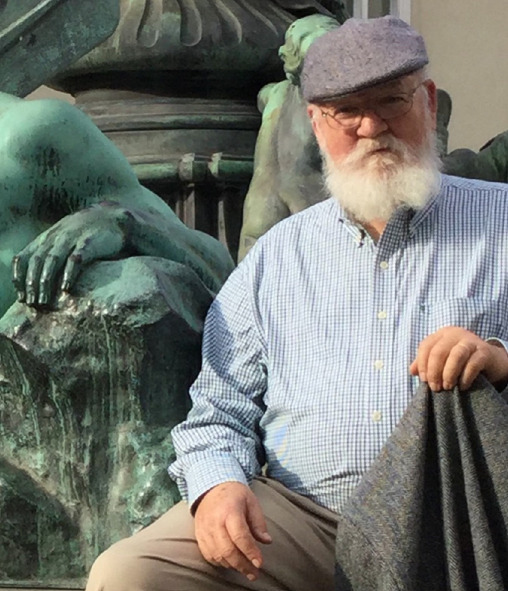


This special issue is dedicated to the memory of Professor Daniel C Dennett, a pioneer of consciousness studies, and a treasured friend and mentor.

## 1. Introduction

Philosophers and neuroscientists studying consciousness have mostly avoided asking a central question: why did consciousness evolve? Assuming that some species (e.g. humans) have consciousness and others (e.g. oak trees or sponges) do not, what problem(s) did consciousness evolve to solve? From a biological viewpoint, and regardless of which species have consciousness (or to what degree), this question of the adaptive function(s) of consciousness is central to an evolutionary viewpoint. Nonetheless, the growing discipline of consciousness studies has largely (but not entirely) skirted this issue.

Our goal with this special issue is to summarize and consider different proposals about the evolutionary function(s) of consciousness from an empirical viewpoint. We argue that tractable and testable proposals about the evolution of consciousness should occupy centre stage in its study, and that a strong focus in the current literature on intrinsically subjective questions about human consciousness (qualia and the so-called ‘hard problem’) have tended to derail attention from these more promising approaches.

Our foundational principle is that the scientific study of consciousness and its biological basis must be empirical and will profit greatly from a comparative and evolutionary perspective. Most current writing on consciousness focuses on humans, frequently presupposing either that humans (or perhaps our closest relatives) are the only species exhibiting consciousness or that only human subjects allow the study of consciousness to be empirically tractable. However, regardless of where one draws the line (at humans, primates, mammals, vertebrates, all metazoans...), hypotheses about the adaptive functions of consciousness remain central to empirical inquiry.

If consciousness has a function, then it should be possible to evaluate whether individuals of a given species can carry out that function or not, and under what circumstances they do so. For example, given the pervasiveness of sleep in bilaterian animals, we could start by asking *when* is an individual conscious or not (whether asleep and dreaming or awake and active), how the functional profiles of dreams and active states differ, and then *what mechanisms* support these conscious states. Similarly, if certain functions of consciousness require particular types of neural circuitry, this should yield testable claims about which species have the relevant circuitry, either examining homologous mechanisms (e.g. cortical recurrence in mammals) or convergently evolved neural mechanisms (as in species that lack neocortex, for example, birds, octopus or insects). The convergence question can only be rigorously approached given a well-defined *functional* account of consciousness.

The editors and authors of this special issue share the view that consciousness can be studied empirically and should be grounded in biological terms, including neurobiological mechanisms, phylogenetic analysis and adaptive function. Despite long-running debates about consciousness among philosophers of mind and an ever-growing engagement by neuroscientists, foundational principles of evolutionary biology are too rarely deployed in contemporary consciousness studies. By treating comparative data as irrelevant or ignoring questions about the functions of consciousness that might be shared among humans and other animals, the literature often presupposes a human exceptionalist viewpoint (though this is fortunately beginning to change: [1,2]). When questions of animal consciousness are raised, arguments often involve opinions about a species' perceived similarity to humans, and personal taste often determines where any given scholar draws the line.

By bringing together and evaluating current opinion on the functions and evolution of consciousness, from the biological perspective, our goal is to enhance the study of consciousness and broaden its appeal to biologists and other scientists who might not otherwise consider these issues. We hope to enrich consciousness studies by illustrating the virtues of the biological approach and to enrich biology and neuroscience by demonstrating the use of functional hypotheses in empirical research programmes. We intend the issue to spark debate, both within and beyond these disciplines.

There are several areas where this evolutionarily and mechanistically informed debate has broader implications for society. One is animal welfare, where poorly grounded pre-theoretic notions about animal consciousness currently have wide ramifications for our treatment of nonhuman animals. For example, the ‘perceived similarity to humans’ approach leads to mammals being accorded greater protection than birds, and fishes having very few protections indeed (often based on the unsubstantiated notion that ‘fishes cannot feel pain’). The second area concerns current debate about consciousness in artificial intelligence and its implications. Should ChatGPT have rights? Can a large language model suffer abuse (or be a co-author)? Third, there are multiple issues in medicine, ranging from the use of anaesthesia to treatment decisions about patients with brain damage, some of whom have been identified as having preserved awareness and cognitive function (i.e. ‘cognitive motor dissociation’) despite being behaviourally unresponsive, that would be greatly influenced by more concrete functional models of consciousness. These issues will both be impacted by the debates in, and conclusions of, our special issue.

## 2. Terminological issues in consciousness studies: what are we trying to explain?

### (i) Multiple facets of consciousness

Most discussions of the biology or neuroscience of consciousness begin by distinguishing different phenomena or aspects of consciousness picked out by the term, prior to singling out one or a few for more detailed consideration. This is doubly true because different aspects of consciousness probably implicate different neurobiological systems. For example, a neuroscientist interested in the distinction between sleeping and waking states will focus on different aspects of neural function (and perhaps will study different organisms) from one interested in the distinction between awareness of attended and unattended stimuli. The wide agreement that there *are* different phenomena referred to by the term ‘consciousness’ has important and immediate implications for our central question in this volume, since if there are multiple phenomena identified they will probably have different functions. So, for example, the state of consciousness in sleep may have a function in promoting memory consolidation, while selective attention may serve to guide appropriate action. Thus, we should be explicit about what we mean by ‘consciousness’ when discussing any functional hypothesis.

This seemingly obvious methodological point is complicated by the fact that philosophers have been discussing and debating the nature of consciousness for centuries, and there is yet no single agreed-upon taxonomy. Indeed, many of the distinctions that have been offered crosscut each other. Even restricting our discussion to the last few decades leaves a plethora of terms and distinctions, sometimes accompanied by subtle book-length arguments about the central relevance and correctness of this or that construct. This leaves empirical scientists little option, practically speaking, other than to adopt some pre-existing term for the type of consciousness they intend to study, cite the appropriate philosophical literature and get on with their experiments. The authors in this collection were free to define or delimit consciousness as they saw fit, and as a result not all use the term in the same way.

That said, we begin by pointing out a few distinctions that are widely recognized. One concerns global states of consciousness, such as waking versus sleeping, which involve different levels of arousal and awareness. Another concerns the subjective or qualitative nature of experience. Most often termed phenomenal consciousness, this is the subjective, first-person, ‘what it’s like’ quality characterizing our waking lives. The experienced subjective character of objects and events, which is the central feature of phenomenal consciousness, has been termed ‘qualia’ by philosophers. The bulk of the papers in this issue are concerned at least in part with phenomenal consciousness. In the philosophical literature, phenomenal consciousness is often contrasted with another kind or aspect of consciousness, ‘access consciousness’ which entails that the contents of consciousness are widely accessible to various cognitive faculties, and (in humans at least) are reportable [3,4].

This conceptual distinction between phenomenal and access consciousness informs many of the more prominent theories of consciousness, such as global neuronal workspace theory (GNWT) [5], recurrent processing theory (RPT) [6] and integrated information theory (IIT) [7]. Note, however, that the distinction between phenomenal and access consciousness is a conceptual distinction: they may be two aspects of a single biological phenomenon. It remains an open question whether the bounds of one can outstrip the other (see [3]). The property of having phenomenal consciousness is sometimes called ‘sentience’, although occasionally the term sentience gestures at something more basic, such as the ability to sense the positive or negative valence of the organism’s own states.

Another distinction that appears many times in the contributions to this special issue is between awareness of a stimulus or object in the external world versus awareness of one’s self. Both sensory consciousness of external objects (exteroceptive consciousness) and of internal bodily signals (interoceptive consciousness) are types of first-order consciousness. An organism’s explicit awareness of its own mental states takes conscious states as its object, thus is considered ‘higher-order’ consciousness. There is little agreement about what is required for awareness of self. As this collection makes clear, different researchers mean different things by self-awareness. Some view it as quite basic, perhaps identical with the notion of sentience. If indeed the function of self-awareness is important for basic survival or for sociality, it is probably shared by many species besides humans. Humans probably enjoy multiple types of self-awareness, including one (or several) that involves having a concept of self. To what extent our self-awareness involves an explicit representation of self is a matter of debate. Whether this is language-dependent is arguable, but a lack of sophisticated cognitive representations has provided grounds for denying (some) nonhuman animals self-consciousness. Some of the papers in this volume address this issue, arguing that consciousness of self can be grounded and evidenced by less demanding and anthropocentric cognitive abilities. This is only a brief sketch of the variety of concepts the term ‘consciousness’ has been used to refer to. Our focus here is what their functions are, and how they are grounded in the (neuro)biology of organisms.

The strategy of defining one’s target before fully understanding it, while understandable, carries considerable risk, because terminology is not innocent. Terms and distinctions are often based on pre-theoretic intuitions and introspection, not grounded in biology or neuroscience. Focusing on a particular conceptual distinction to pick out a particular phenomenon of consciousness provides a reasonable strategy for embarking on a research programme, but such concepts may or may not ultimately map onto functional, neurobiological or mechanistic explanations in the right way. We should thus remain open to revising our concepts in light of new data and new theory, but not so open-minded that no critical standards apply.

Undoubtedly, the warning above concerning terminology applies to psychological as much as philosophical terms, but in psychology the empirical and experimental foundations provide more cause for optimism. Commonplace distinctions like implicit versus explicit learning, or procedural versus declarative knowledge, originate in human experimental psychology. Because they rely on self-report, they are quick and seemingly easy distinctions to make (in adults): if the experimental subject can state the rule that was learnt, it involved explicit learning; if not, it was implicitly learnt. Some knowledge or ability that can be expressed motorically (e.g. riding a bike) but cannot be explicated verbally is considered ‘procedural’ knowledge, while verbally expressible facts (‘Paris is the capital of France’) are considered ‘declarative’. Such terminology is fine if you are working with college undergraduates, but far more challenging if we turn to nonhuman animals or pre-verbal infants.

Indeed, any distinction based on linguistic self-report seems *de facto* inapplicable to non-linguistic organisms, since they can never ‘state’ what they have learnt and know. However, these terms are nonetheless used in comparative psychology and neuroscience, with the following adaptation. If a rat or monkey has been trained to press a lever for some rewarded class of stimuli (S+), and not press for some other class (S−), we can consider the trained animal’s lever presses as a form of self-report, and despite some etymological slippage, extend the term ‘declarative’ to such responses, which are considered to be conscious and known to the animal. For example, monkeys have been trained to hold a button when seeing an image of any face. When such a trained monkey is now exposed to binocular rivalry stimuli (where images of a face and house are presented in the same spatial location, but to different eyes), they will sometimes hold the button, then release it, and then press again. Humans presented with the same stimuli self-report that their perception cycles between seeing the face and seeing the house; this is a classic bi-stable stimulus. Usefully, we can then perform brain imaging in humans and even combine this with single-unit recording in the monkeys, to determine whether similar neural activations underlie this cycling in the two species: and they do [8,9].

A host of different experimental paradigms have been used in animals that have extended the procedural/declarative distinction to mice and rats, and a general conclusion is that hippocampal and temporal circuits play a key role in ‘declarative’ learning, while basal ganglia and motor circuits play a distinct role in procedural learning. These results are congruent with data from human clinical neuroscience, where for example damage to hippocampus and surrounding regions leads to a profound inability to ‘remember’ new experiences (in the sense of verbal report) with a spared capacity for procedural learning of new actions [10]. In a sense, despite retaining the original human-oriented terms, this branch of cognitive neuroscience has begun to replace the human-oriented concepts, based on self report, with a more behaviourally and neurally based conceptual framework, greatly extending its scope and explanatory power.

By analogy, we may hope that the consciousness scientists of the future can confidently distinguish between, say, brainstem, thalamic and pallial or cortical contributions to different forms of consciousnesses, and understand their underlying algorithmic/adaptive function, their subjective correlates and their neural signatures. Furthermore, future biologists will hopefully be able to pinpoint where, when and why these distinct forms of consciousness are found in other species. Alas, we are not there yet, but the contributions in this volume, by offering concrete hypotheses about the functions of consciousness, are first steps in this direction.

### (ii) A cautionary tale: qualia and philosophical zombies

We have cautioned that choice of terminology may smuggle in preconceptions about the object of study that are misleading or false. A case in point again involves the term ‘qualia’, which has played a central role in many recent discussions of the neuroscience of consciousness. Qualia (singular ‘quale’) are supposed to pick out those aspects of a perception/experience that are private, subjective and qualitative: the redness of red or the painfulness of pain. Nagel and others have compellingly argued that the subjective nature of experience seems independent from ‘objective’ aspects of an experience, for example the electromagnetic wavelength reflected by a coloured stimulus, or increased cone activity and neural firing in visual cortex accompanying (and presumably causally contributing to) its perception.

Based on this subjective/objective distinction, neuroscientists seeking the neural correlates of consciousness often characterize their enterprise as seeking that subset of neural processes that support, enable or underlie ‘qualia’: the personal, phenomenological side of perception. Philosopher David Chalmers famously distinguished between ‘easy’ and ‘hard’ problems of consciousness in these terms. The ‘easy problem’ concerns all the aspects of consciousness studies that attempt to explain the functional aspects of consciousness: the ability to distinguish colours, direct attention, recall memories and so on, in objective, neuroscientific terms of third-party observables and data. However, Chalmers argues that this study, however useful and successful, can never meet the real challenge: explaining the subjective, first-person ‘what it is likeness’ to experience red, or pain, or have any other conscious experience whatsoever [11]. This is what Chalmers dubbed the ‘hard problem of consciousness’, and despite numerous critiques (cf. [12]), this framing has played a central role in contemporary discussions of consciousness and its study. However, note that the idea that you can fractionate problems of consciousness into separable functional and phenomenal problems implies that the phenomenal story has no functional basis.

The separation between the functional and phenomenal is based on a thought experiment, and an intuition sometimes called the ‘zombic hunch’. We are asked to entertain the idea that an organism could behave exactly like a normal conscious being, while in fact entirely lacking phenomenal consciousness. Such imaginary creatures are dubbed (philosophical) zombies. Imagine, for example, a zombie organism with all the neural circuitry required to both register tissue damage and reflexively pull away from the source of damage, but, lacking subjective experience entirely, would be otherwise indifferent to the normally pain-causing stimulus. In human form, this zombie could even exclaim: ‘Ouch! that hurt!’ and presumably convince those around it that it was conscious, despite, according to this thought experiment, feeling nothing at all at a personal, subjective level. Indeed, carrying this thought experiment to its conclusion, the entire inner world of zombies would be empty. There would be nothing ‘it is like’ to be a zombie, because there’s ‘no one home’ inside. Chalmers has argued that our very capacity to entertain this argument, to imagine philosophical zombies, is *prima facie* evidence that the phenomenal and functional are conceptually distinct, and that consciousness cannot thereby be explained by functional stories.

One might cast the long history of philosophers denying consciousness to nonhuman animals in these terms. What is the ‘special sauce’ that endows us with subjective experience, making us non-zombies? Candidates abound: God, the soul, language or von Economo neurons are just some of the candidates that have been offered. Indeed, although we do not endorse this perspective, one could conceive of phylogenetic inquiries about the origin of consciousness as asking ‘when in evolution did organisms cease being zombies, and what advantages for survival and reproduction did non-zombiehood bring?’ However, note that since, by the zombie hypothesis, there are no behavioural differences between humans and philosophical zombies, qualia cannot provide any additional adaptive benefits to the organisms that have them. Being behaviourally inert they would seem to be biologically epiphenomenal.

In this sense, even asking the central question of this volume—what are the functions of consciousness?—potentially undercuts the intuition driving zombie-based arguments. If *any* unique purpose or (non-redundant) function of qualia exists, the zombie hunch is falsified: an organism lacking qualia would also be incapable of executing that function and any behaviours that require it. Moreover, for a function to evolve, it would need to have such behavioural readouts. Natural selection only acts on external sequelae of cognition and is blind to any posited inert inner contents. Thus, if we adopt a standard notion of adaptive functions as referring to those which result from the action of natural selection, any convincing adaptive function of qualitative consciousness immediately calls the ‘zombie hunch’ into question, along with many of the conclusions that some philosophers have confidently drawn from zombie-based arguments. Returning to pain, if the subjective ‘painfulness’ of pain (its ‘qualia’) plays a necessary role in influencing the organism’s future actions, e.g. in learnt avoidance of the place or object where the pain was experienced in the future, a zombie lacking that feeling would, despite an initial appearance of pain-aware behaviour, also lack those behavioural sequelae.

Undoubtedly, our question about the adaptive functions of qualitative consciousness could be seen as begging the question raised by zombie advocates, for the zombie thought experiment is meant to show the conceptual independence of qualia from behaviour and neurology. It seems to us, however, that siding with the zombies is throwing in the towel too soon. As the articles in this issue illustrate, there are plausible stories about how and why subjectivity arises, and even if we lack a compelling mechanistic story explaining it now, we are willing to bet that as science develops, things that previously seemed forever inexplicable will begin to yield their secrets. We take the mystery of consciousness to be similar to the mystery of life. A few centuries ago, it was thought that life could not be explained by any material process—it was so mysterious it had to be owing to a divine spark, or *élan vital*. However, as science progressed, we began to recognize that life was rather a complex constellation of processes, and that these could be mechanistically explained. This is another reason why defining consciousness too early may mislead—we may not yet know enough to know how to fully or accurately define or describe its various features and their functions. The characteristics of consciousness, like many other physical concepts, such as mass, temperature, or heat, or biological concepts like ‘gene’ or ‘chromosome’, may only come to be properly understood during the process of investigation, as measurements become more refined and reliable [13,14]. As with the concept of life, we may look back one day and realize that what we did not know prevented us from seeing how consciousness could and should be explained.

## 3. Towards a biologically grounded taxonomy of consciousness

A promising start for a new conceptual framework for evolutionary approaches to consciousness is to follow Tinbergen’s famous injunction to ask multiple ‘why’ questions from different biological perspectives [15]. Now enshrined as Tinbergen’s ‘four questions’, these include answering mechanistic questions (e.g. concerning the neurobiological basis of consciousness), adaptive or ‘functional’ questions (concerning ultimate adaptive value: ‘what for?’), phylogenetic questions (when, how, and in which species, did consciousness evolve: ‘how come?’) and ontogenetic questions (when, during individual development, does consciousness arise). Tinbergen’s insight, now widely accepted by biologists [16,17], was that solid understanding of biological, and particularly behavioural, traits requires answers to all of these questions, answers that are both internally consistent and that mutually inform one another [18]. Thus, rather than focusing solely upon (say) mechanistic or adaptive explanations (or worse, seeing these as being in competition), we should study both and integrate the answers. In particular, mechanistic or ontogenetic understanding, at the individual or ‘proximate’ level, can anchor and inform our explanations of ‘ultimate’ functional and phylogenetic questions.

To choose a straightforward example, mechanistic models positing that consciousness is realized in neocortex directly imply that non-mammalian organisms lacking neocortex also lack consciousness (because neocortex is a novel neural tissue type, found only in mammals [19]). By contrast, models that situate basic consciousness in the basal forebrain and brainstem (e.g. [20,21]) would extend it to most or all other vertebrates, given the deep conservation of fundamental brainstem neuroanatomy and neurochemistry across all vertebrates (including fishes, frogs, birds and reptiles: [19,22]). Alternative, more computational and substrate-neutral frameworks focus on one or more core information-processing functions that underlie consciousness. For example, while neocortex may provide the substrate for information integration in mammals, the same role is played by different circuits in birds, which lack neocortex but accomplish similar functions with their mostly unlayered, nuclear telencephalon [23]. Given the clear interconnectedness between mechanism and function, it is unsurprising (and consistent with Tinbergen’s advice) that many of the contributions in this issue delve deeply into the neurobiology of consciousness.

### (i) Multiple notions of biological function

Turning to function *per se*, it is first important to note the polysemy of the term ‘function’, which sometimes refers to a specific mechanistic (‘engineering’, or what/how) function, in an individual, and sometimes to the broader adaptive (why) function of some trait in a species, that explains why the trait spread through a population (often distinguished as ‘proximate’ and ‘ultimate’ functions, respectively, following [24]). For example, the proximate engineering function of the heart is to pump blood, but determining its ultimate adaptive function may require reaching far back in evolution to a time in which we have only limited evidence about extinct species. The evolution of hearts from pulsatile tubing probably supported more efficient delivery of oxygen and nutrients in early bilaterians more than half a billion years ago, leading eventually a dedicated closed circulatory system [25]. For our purposes here, it is not crucial to distinguish between these different notions of function at the outset: either category will serve as *a* function of consciousness, worthy of further empirical investigation and conceptual refinement, even though the evidence base similarly ranges from analysis of the behaviour and brains of current organisms to more speculative claims about the behaviour and nervous systems of long-extinct species.

To illustrate some of the complexities lurking in the concept of ‘biological function’ we will choose a relatively uncontroversial example: the function of feathers in the powered flight of birds. First, note that ‘flying’ is a general term, covering a diverse set of both artificial (airplanes or helicopters) and natural (bird, bat or insect flight) examples. What all of these systems have in common is a self-powered capacity to move a solid body through the Earth’s atmosphere, potentially countering the force of gravity. Neither a falling object nor a thrown object have this capacity: gliding is not considered flying under this characterization, nor would metaphoric extensions (penguins are not flying underwater, nor are an airplane passenger or a bat’s flea flying). Despite the specificity of this definition, the diversity of flying systems would render any attempt at a fully general engineering analysis of flight rather unrevealing: a general theory covering both helicopters and bats would entail that certain principles of physics and aerodynamics be obeyed, but little more than this. Similarly, there are equally numerous possible purposes or functions of flight, from migrating or capturing food to making money or dropping bombs.

However, if we consider instead the functions of *feathers* in flight, we quickly engage in a productive research programme (and for this particular case, one where all of Tinbergen’s questions have been asked and at least partially answered (cf. [26,27])). First, in any living bird, we can observe that there are multiple types of feathers with distinct functions: insulating down for retaining heat, shedding water, coloured crest or plume feathers for sexual displays, or even specialized sound producing feathers [28], and only wing feathers are specialized for their function in flight. Flight feathers have particular characteristics (e.g. asymmetry and curvature) that solve engineering problems like efficiently generating lift and avoiding turbulence. Furthermore, the fossil record makes clear that feathers evolved *before* powered flight (in feathered dinosaurs), and numerous secondarily flightless birds that retain feathers (e.g. ostriches, kiwis or penguins) indicate that the phylogenetic history of feathers is complex and involves multiple changes of function [26]. We thus need to distinguish between past and present functions. However, turning to the pennaceous feathers used for powered flight in most birds, there are a host of specific morphological features that suit their current function in powered flight (e.g. morphological asymmetry of the feather for power, tubular nature for light weight, asymmetrical protein arrangement for springiness, etc. [29]). Feathers are also integrated into both wing morphology and the whole organism to make efficient powered flight possible: feathers alone do not fly. Functional analyses of feather evolution take all of these (and other) considerations into account [27].

A central conceptual distinction in discussions of biological function is between ‘selected effects’ or adaptive functions (what philosophers term ‘etiological’ functions: [30–32]) and current mechanistic or ‘causal role’ functions [33], both of which play central roles in biological explanation [34]. Physiologists tend to study current causal functions, and palaeontologists the origins and evolutionary history of adaptive functions. These two interpretations of ‘function’ are often but not always related [35]. For an ostrich (a flightless bird whose ancestors had flight), the wing feathers no longer fulfil this ‘original’ adaptive function, and their current causal roles may include thermoregulation or display, but do not function in flight. However, for a flying bird, the selected effect of flight feathers and its causal role in flight are closely related, and this relationship warrants solid inferences based on morphology to extinct species (e.g. that the extinct bird *Archaeopteryx* was capable of powered flight). Hence there are several distinct lines of inquiry into biological function, and it is useful to clarify which of these are in focus, and what if any the posited link between current use and past selected function is [16].

By analogy to flying, we suggest that researchers on the functions of consciousness recognize that there are multiple potential functions of consciousness—both in terms of current use and past selection—and clarify which aspect(s) they focus on [1,2]. There are already a host of options on the table for these functions of consciousness (for reviews, see [31,36–39]), but these are unlikely to cover all of the options. Furthermore, by analogy to feathers, progress will be accelerated if researchers pick out particular components of consciousness and tie them to potential sub-functions (e.g. the role of valence in decision making in the face of motivational trade-offs [40]. Of course, consciousness science lacks a theoretical framework as solid as Newtonian physics and aerodynamics to ground such discussions, but information theory, computer science and modelling all can play supporting roles in this endeavour. Crucially, just as for flight, comparative data across a range of species can help clarify our thinking and test specific functional hypotheses about consciousness (cf. Jablonka & Ginsburg [41]). In pursuit of a diversity of hypotheses, while preparing this special issue, we did not attempt to direct which type(s) of functions the contributors took as their focus. We think that the diversity of views represented here speaks for itself, and provides ample fuel for future researchers into the evolution of consciousness.

### (ii) Current functional approaches: an overview

Adaptive, functional accounts of phenomenal consciousness change the focus from the subjective ‘how it feels’ to the objective behavioural readouts of such feelings. In a famous paper, philosopher Thomas Nagel asked ‘what is it like to be a bat?’ and concluded that we might never know the answer, owing to the intrinsically subjective and first-personal nature of qualitative experience [42]. Answering this question, which is closely related to Chalmers' ‘hard problem’, remains a major challenge and preoccupation of consciousness research today. However, accepting that some aspect of mental processing leads a subset of our cognitive processes to ‘become conscious’ in this sense, philosopher Daniel Dennett pointed out that an equally important but neglected question is ‘then what happens?’ [12]. In other words, what are the sequelae of this internal, subjectively experienced cognitive event on our subsequent objective behaviour? One goal of many of the articles in this issue is to grapple with this central functional question. Dennett himself termed this the ‘hard question’ of consciousness (to contrast it with Chalmer’s ‘hard problem’), but in his honour we might term this question of the functions of consciousness ‘*Dennett’s question*’. Asking Dennett’s question, as the authors in this volume do, does not entail ignoring Nagel’s ‘what is it like’ question—in true Tinbergian fashion, both are important and relevant, and we need answers to both. However, only when we understand how the internal subjective aspects of our experiences impact our actual behaviour will we understand why consciousness evolved, and why it exists in some species but not others.

It is important to note that functional arguments regarding consciousness do not entail that all aspects of consciousness are adaptive all of the time (cf. Tramacere [43]). It suffices that functional aspects of consciousness are functional on average, and in the long run function better than unconscious variants of the same type of processing. For example, the fact that we occasionally experience visual illusions clearly does not imply that vision is dysfunctional. Nor do longer-term disturbances of consciousness, like schizophrenia or dementia, imply that normal non-clinical consciousness is somehow maladaptive or epiphenomenal. Indeed, the clearly dysfunctional behavioural readouts of disturbances of consciousness is *prima facie* evidence that (phenomenal) consciousness *has* function(s).

We will not try here to summarize all the extant hypotheses of the functions of consciousness (for concise reviews see [31,37–39]). Many of these (but not all) are discussed in detail by the authors of this special issue. However, it may be useful to provide a general overview of the types of hypothesis on offer before providing a more detailed summary of those presented here. There is a widespread agreement by many commentators on this problem that consciousness serves to ‘increase flexibility’ of cognition (e.g. [44–46])—a general viewpoint that has been termed the ‘integration consensus’ [47, p. 1002]. However, most of the authors here try to provide a more specific account, and it may well be that many of these are correct. For example, the widely cited hypothesis of Ginsburg and Jablonka that phenomenal consciousness supports a generalized form of associative learning that they term ‘unlimited associative learning’, posits a future-oriented, *prospective view* of the function of consciousness [1,2,48–50]. The capacities to learn exhibited by different organisms provide an objective readout of such prospective functions.

By contrast, other hypotheses see a key role of consciousness as deciding between mutual competing options *in the moment*, depending on current context and current needs. An organism might be thirsty, hungry and threatened by a predator simultaneously—what should it do, right now? Following the lead of Michel Cabanac [51], Brown & Birch [40] suggest that the valence of the various competing drives acts as a common currency for reaching such real-time decisions. Selective attention would be another current, real-time function: given competing stimuli, which to attend to? (see Cabral-Calderin [18]). Humphrey’s suggestion [52] that the core purpose of our conscious experience is to make experiences matter, to us as individuals, would also fall into this category (though Humphrey, following William James, sees this as extending across a small but important timespan he terms the ‘thick moment’). Tramacere [43] suggests that in-the-moment expansion or contraction of subjective, experienced time serves as an ‘efficiency amplifier’, providing more or less cognitive processing power, as needed. As Brown and Birch suggest, experimental investigations of motivational trade-offs provide an excellent way to probe such real-time functions of consciousness in a wide variety of organisms.

Finally, a third category of function is *retrospective*—that consciousness is about knitting together a subset of past experience into a coherent ‘experienced present’. This perspective on conscious experience was championed by Daniel Dennett [53–55] and though controversial when first proposed, it has become increasingly widely accepted (e.g. [4,6]). Crucially, these different temporal aspects of time are not mutually exclusive. For example, Fitch has argued elsewhere that the function of the ‘seriality’ of consciousness exists to retrospectively broadcast action decisions and their outcomes to all circuits relevant to reaching the decision, in order to prospectively allow allocation of credit or blame during learning [56,57]. This functional hypothesis thus combines retrospective and prospective functions. It seems likely to us that many of these proposed functions will turn out to be correct explanations of different facets of consciousness (also probably underpinned by different neural computations). Our hope in this volume is to put a variety of possible functions on the table, not as any final hypothesis set, but as a provisional basis for further exploration.

## 4. Brief introductions to the articles in the special issue

The contributors to the proposed special issue have been chosen specifically for their interest in and writings about the evolutionary functions of consciousness. The authors come from quite different backgrounds including neuroscience, philosophy and cognitive biology, but they are unified by their interest and expertise about this core question. In prior contributions, scattered across a wide disciplinary variety of journals and books, these authors often advocate some specific function or mechanism for consciousness, but we know of no single place where such different viewpoints are brought together as a basis for further discussion and evaluation. Our main goal in putting together the special issue was to provide an overview of these many possibilities and ignite interest in further conversation and debate.

In what follows we provide a brief overview of the articles in the special issue, which we have grouped for convenience into three categories of five to six articles each: ‘adaptive perspectives’, ‘behavioural readouts’ and ‘neurocomputational perspectives’. We recognize that any such compartmentalization has some Procrustean elements, given that many of the articles touch on multiple themes. Nonetheless, combined with the brief summaries below, this categorization should aid readers in seeking out the articles most pertinent to their own interests.

### (i) Adaptive perspectives: proximate and ultimate functions

Irina Mikhalevich [58] provides a brief historical review of the study of animal consciousness and its evolution and highlights three key problems that make the ‘new naturalism’ pursued by current researchers a significant challenge. First, she explores the issues surrounding evolutionary explanation in general, and particularly the idea that consciousness is an adaptation (versus a byproduct or exaptation of something else), arguing that this issue is more subtle and problematic than often thought. Second, she highlights the measurement problem: we have few agreed-upon tests that unequivocally indicate that a particular species has consciousness (or some subtype of consciousness, such as phenomenal consciousness or awareness of valence). Third, she confronts the problem of ‘trait individuation’, concerning how to devise and improve a taxonomy of subtypes of consciousness that is not intrinsically anthropomorphic. Together, she argues, these three problems are inter-related in important ways, and pose unique problems that need to be clearly acknowledged (while being no cause for defeatism) by researchers in animal consciousness. Finally, she explores these three problems by contrasting adaptive accounts of phenomenal consciousness (aka ‘sensory consciousness’) versus valence-oriented accounts (aka ‘affective consciousness’), showing how it is conceivable that one of these could be adaptive *sensu stricto* and the other a byproduct of it. Resolving this issue requires that we clearly articulate the causal relationship among these different aspects of consciousness and seriously consider and contrast adaptive and byproduct explanations for different aspects of consciousness.

Eva Jablonka and Simona Ginsburg (JG) [41] build on a decades-long theoretical investigation of consciousness, in which they have argued that a form of nearly *unlimited associative learning* (UAL) integrating multiple sensory modalities, short- and long-term memory, valence and agency, provides a clear, empirical ‘marker’ of what they have called ‘basic’ or ‘minimal’ phenomenal consciousness [1,2,48,49]. They suggest that, although UAL is not found in all animals (being present in all vertebrates, some arthropods, and cephalopod molluscs), it appeared early in evolution during the Cambrian and has evolved convergently several times. After providing a primer on, and further references for, UAL theory, they argue here that consciousness creates a new category of selection: ‘mental selection’. They note a curious lacuna in evolutionary theory: despite a huge literature distinguishing between different varieties of selection (sexual versus natural selection, r- versus k-selection, individual versus group selection, to name a few), few theorists have singled out the potential selective effects of mental states, such as goals and desires, in evolution. JG set out to fill this gap, arguing that mental selection by conscious organisms has important and pervasive effects on all life. Mental selection is intermediate between mindless natural selection and the rational, conscious selection Darwin termed ‘artificial selection’ (e.g. by humans during recent plant and animal domestication). They illustrate the role of mental selection by considering the evolution of camouflage in predator–prey interactions, and of signal design in mating displays, arguing in support of Darwin’s belief that such signals, particularly signals selected by ‘choosy’ mates, inevitably reflect the mental powers of the signal recipients. They conclude that evolutionary theory remains incomplete as long as it neglects the role of conscious cognition in evolution.

Nicholas Humphrey [52] summarizes his 50+ years of scientific exploration of phenomenal consciousness, relying heavily on his research on *blindsight* [59]. Blindsight is a peculiar phenomenon where human patients with lesions to visual cortex deny awareness of stimuli in the affected visual field but are nonetheless able to respond behaviourally to such stimuli [60]. Blindsight can be experimentally induced in monkeys via surgical ablation of visual cortex, and Humphrey relates his personal experience with a macaque who had undergone this procedure, ‘Helen’. Humphrey was able to demonstrate a rather high level of visually guided behaviour in this monkey, presumably guided entirely by subcortical visual processing, despite apparent remaining deficits in her phenomenal awareness of visual stimuli. Similar high-level visual functioning, including high acuity and attention-guided responses, has also been demonstrated in human blindsight patients. These data lead Humphrey to suggest a distinction between ‘*cognitive consciousness*’—the ability to process visual input and integrate it at a relatively high level—and phenomenal consciousness of the sort experienced by intact humans or monkeys. Humphrey provides an evolutionary model for how and why phenomenal consciousness arose via the internalization and ‘virtualization’ of reflexive responses to stimuli via efferent copies and suggests that this is the exclusive purview of homeothermic vertebrates (birds and mammals). As to its adaptive function, he builds on his past arguments [61] that it is crucial that these internalized reactions *matter* to us, as individuals, or else they could be easily ignored or overlooked (a suggestion further explored later in the issue by Moncoucy and colleagues [62]). In summary, Humphrey offers an integrated evolutionary functional account of phenomenal consciousness, tied closely to neuroscientific findings.

Léa Moncoucy, Krzysztof *Dolega*, Catherine Tallon-Baudry and Axel Cleeremans (MDTC) [62] also treat phenomenal consciousness within a learning framework, proposing an evolutionary trajectory in which phenomenal experiences transitioned from being proxy signals for extrinsic use to intrinsic evaluations that directly motivate decision-making and learning by serving as inputs to cognitive systems. MDTC suggest that the function for which phenomenal consciousness was originally selected is to signal the approximate adaptive value of internal and external states to a subject. However, in taking on the intrinsic valuation role, phenomenal states become themselves the targets of behaviour: organisms are largely unaware of the ultimate reproductive or survival value of their actions but instead work to achieve proximate pleasurable feelings or remove unpleasant ones. The idea that organisms seek pleasure for its own sake and avoid pain likewise is a longstanding idea, of course. However, MDTC propose that similar to sexual selection the emergence of phenomenal states fuelled a runaway evolutionary process in which new kinds of behaviour and more sophisticated kinds of control mechanisms persisted despite being sometimes detrimental to the more fundamental biological imperative to survive and reproduce. They take a more expansive view of the relevance of phenomenal consciousness to learning than Jablonka and Ginsberg. They cite research on human subjects by Skora and colleagues to support the idea that even relatively limited forms of learning require consciousness. The link to instrumental learning suggests directions for empirical research to take, but specificity about the mechanisms involved seems necessary.

Kristin Andrews and Noam Miller (AM) [63] present the novel hypothesis that the original function driving the evolution of sentience is *sociality*. They remain silent on the initial emergence of sentience but argue that it became a target of selection for organisms needing to solve problems of social coordination. The Cambrian explosion led to animals engaging in much more variable and less predictable ways. AM argue that if the benefits of group living are to be maintained in this context, individual organisms must pay more attention to the behaviour of others. Initially, separation from conspecifics induced negative affect and integration with the group produced positive affect, and these affective states were used along with better behavioural prediction of others to support synchronized and coordinated behaviour. AM take three lines of evidence to support their account: (i) the widespread distribution of consciousness, arguing for an early evolutionary origin; (ii) the tight neurological integration between social cognition and affective processing; and (iii) the priority given to removing social pains even at the cost of bodily pain in a wide range of organisms. AM suggest several avenues for empirical investigation of this hypothesis in nonhuman animals, including testing the saliency of social stimuli for attention and testing for over-attribution of agency, and they advocate for a shift from focusing on the cognitive sophistication of animals to a focus on the kinds of stimuli driving their behaviour.

Although consciousness is sometimes seen in binary terms—either you have it or you do not—many articles in the current issue point out that consciousness is graded. In a novel take on this idea, Antonella Tramacere[43] focuses on a well-known but little-studied aspect of consciousness: *time distortion*. During dangerous or highly challenging events, time seems to slow down. This phenomenon of subjective ‘time dilation’ is typically reported during accidents or other life-threatening events. The opposite effect of ‘time constriction’ is seen during routine, automatized action, and is also observed experimentally when one event (e.g. pressing a button) is perceived as causing another (e.g. a bell ringing). During such volitional, intentional actions, the delay between action and response—between cause and effect—is perceived as shorter than during other, non-causally related, actions (64). Crucially, reaction times remain undisturbed during such subjective distortions of time, leading to Tramacere’s hypothesis that by increasing subjective duration while preserving normal reaction times, time dilation provides additional virtual time: by increasing its ‘clock speed’ the mind gains additional cycles during which to act adaptively. By contrast, time constriction decreases cognitive load for predicted actions, saving processing cycles for other aspects of cognition. Tramacere thus suggests that both directions of time distortion serve the same overall function: they amplify cognitive efficiency in ways that are fundamentally adaptive. Crucially, both humans and other animals are capable of reporting these subjective changes, making this an ideal and unexplored route to explore phenomenal consciousness in other species. By anchoring her hypothesis in neurocomputational terms (in terms of coding efficiency) and long-standing findings from operant conditioning research and offering experimental paradigms to further test its predictions in nonhuman animals, Tramacere opens an exciting new door for comparative exploration of consciousness.

### (ii) Behavioural readouts of consciousness

In the first piece in this section, Yuranny Cabral-Calderin, Julio Hechavarria and Lucia Melloni (CHM) [18] propose shifting the driving question in consciousness research from *who* is conscious, to *how* and *why* consciousness manifests across the animal kingdom. They advocate a neuroethological approach to consciousness studies centred on Tinbergen’s four questions. These questions concern the neural mechanisms, the function, the ontogeny or development within species, and the evolution across species, of aspects of consciousness. In particular they propose starting with human-centred examples of consciousness, but then suggest expanding beyond traditional paradigms with comparative approaches that take into account the way in which conscious states in humans might vary at different stages of development or given different ecological contexts, and then extending these questions to address the special problems and affordances that other species have given their ecological niches, and given evolutionary relationships. They sketch what this could look like given the example of *selective attention*. The comparative, multi-species framework they describe offers a powerful foundation for developing a robust, biologically grounded theory of consciousness.

Simon Brown and Jonathan Birch (BB) [40] explore the role of valence in adaptively resolving *motivational trade-offs*. They note that the role of trade-offs in ‘normal’ biological adaptation is well-known, and that natural selection can account for the relatively simple forms of trade-offs observed in bacterial chemotaxis, for example, without requiring any attribution of consciousness to bacteria. However, metazoans typically face a suite of multiple competing motivations (e.g. for food, safety, sex or comfort) that must be weighed against multiple aversive stimuli like pain, cold or dangers from predators. Emotional valence is hypothesized to provide a re-representation of stimuli into a common currency that allows flexible, context-dependent decision making. This builds on the idea of Cabanac that ‘valence’—in the sense of the attribution of ‘goodness’ on a simple, positive to negative scale—provides just the sort of common currency that is needed for such comparisons and decisions [51,65]. BB extend this idea with a useful analysis of trade-offs in different organisms. For example, hermit crabs encountering a new shell (with or without a current occupant) must decide to stay in or abandon their old shell, and whether to fight for a new one. Experimental work demonstrates that they do this in highly flexible and context-dependent manner. By contrast, they offer the example of the nematode worm *Caenorhabditis elegans* as approaching trade-offs in a relatively inflexible manner which BB posit does not require phenomenal consciousness—more like bacteria than hermit crabs. Thus, BB offer an overview of an approach to consciousness which makes clear experimentally testable predictions applicable across the animal kingdom.

Jonathan Crystal [66] discusses *episodic memory* as a promising way to experimentally study subjective experience in animals. He provides evidence that in episodic memory experiments, rats remember back in time to their original experience. They show evidence of encoding and retrieving the *what, when* and *where* aspects of experience, of source memory. The data further suggest that rats can replay the temporal flow of past experiences. Since subjective experience is core to episodic memory in humans, and some nonhuman animals show the other canonical aspects of episodic memory, we should infer that they too have subjective experience of such ‘mental time travel’. From a functional viewpoint, the ability for mental time travel, among other things, enables use of information that was not necessarily known to be important at the time of the original encounter. Thus, episodic memory provides a testable candidate function for consciousness and is thus both an important element of evolutionary accounts of consciousness, and a promising behavioural readout of this aspect of subjective experience.

Lars Chittka**,** Sarah Skeels-Jungius, Olga Dyakova and Maxime Janbon (CSDJ) [67] review historical and current efforts to study consciousness and complex cognition in insects. Well over a century ago, insect researchers pioneered discussions of animal consciousness, but CSDJ lament that insect researchers have been overtaken by those working on vertebrates. CSDJ distinguish different aspects of consciousness—sentience, self-recognition, predictive processing and the cycle between sleep and wakefulness—and review a number of recent experiments providing evidence on each of these aspects. While they admit that the topics they review are idiosyncratically chosen, it satisfies their overall goal of showing that methods developed for vertebrates can be applied to insects, and that when this is done, the results often match those for vertebrates. For example, they recount how fruit flies seek out alcohol when deprived of mating opportunities and ingest it despite the fact that doing so reduces their longevity. Similar correspondences between insect behaviour and the behaviour of humans and other animals form the backbone of their general case for insects as model organisms for understanding the evolutionary steps towards consciousness as subjective experience. The overall goal of producing more detailed accounting of the capacities of insects that might be relevant to consciousness is the core of their empirical proposal. They are sceptical of Jablonka and Ginsberg’s view that the function of consciousness is linked to associative learning, but they do accept that felt emotions can enhance learning and they think that some of the evidence from insects is best explained in terms of emotions rather than unconscious motivational states. They also argue that there is a tight link between *prediction errors* and consciousness when the errors involve learnt global patterns and they argue that this is an approach that has not yet been, but should be, pursued with insects.

Masanori Kohda**,** Shumpei Sogawa and Redouan Bshary (KSB) [68] distinguish between a sort of implicit self-awareness that they claim all organisms that move adaptively in the world must have, and an explicit awareness of self. They note that self-awareness of this sort (which they call private self-awareness) is typically diagnosed by the *mirror self-recognition* (MSR) test. Few species other than humans have been clearly documented as passing that test—great apes, dolphins and some corvid species. However, recent results suggest that a coral reef fish species, the cleaner wrasse, also passes the MSR. KSB evaluate the relevance of the MSR test for claims of self-awareness, suggesting that the MSR is highly prone to false negatives (a suggestion further amplified by Maldarelli and Güntürkün [23]). KSB then consider various hypotheses about why and when the capacity for self-recognition arose, suggesting that both brain size and social pressures could independently contribute to the development of the capacity. However, among the potential explanations of the pattern of species that pass MSR, they ultimately favour the idea that self-recognition evolved early on in vertebrate evolution.

### (iii) Neurocomputational perspectives

Our special issue ends with some more detailed explorations of the neural mechanisms postulated to support different aspects of consciousness, and of the degree to which such mechanisms are observed across metazoan phylogeny.

In the first contribution of this section, Gianmarco Maldarelli and Onur Güntürkün (MG) [23] explore the idea that consciousness is likely to be present in many species that are phylogenetically distant from each other, with remarkably different brain structures. In particular, they review the recent literature on consciousness in birds, and argue that many bird species are likely to possess both sensory consciousness and self-awareness. They briefly review three prominent neural theories of consciousness, the GNWT, the RPT and the IIT. They argue that despite not having a cortex, sensory areas of the avian pallium have cortex-like neural organization, and cite evidence that neural activity in a nuclear structure in birds, the nidopallium caudolaterale (NCL), correlates with the animal’s subjective report. Thus, physiological evidence for sensory consciousness in birds exists. Moreover, the data thus far do not discriminate between theories of consciousness: pending further testing, avian data seems consistent with the requirements of all three considered theories. With respect to self-awareness, the avian evidence is less clear-cut. MG further discuss the MSR test, noting that thus far, few birds have passed the standard mark test. However, they again suggest that the mark test is prone to false negatives, often attributable to lack of ecological validity. They note that birds perform other mirror-related actions that suggest self-recognition, such as the ability to discriminate between videos of themselves and conspecifics, or differential reactions to reflections of themselves compared to other birds. These data together suggest that birds know when they see themselves, and distinguish this from a moving image of a conspecific. The evidence discussed in this piece suggests that birds, despite their quite different brains, have both the neural hardware necessary for consciousness and the behavioural readouts thereof.

Joseph LeDoux [69] offers to thread the needle between scepticism about conscious experiences in nonhuman animals and the idea that it is worthwhile to investigate consciousness in at least some animals. Because he takes verbally reportable human consciousness (‘the only kind of consciousness we truly know exists’) as the fixed point against which hypotheses linking anatomy to cognitive and behavioural capacities can be assessed, he refuses to ‘speculate’ about anything other than mammals, whose neuroanatomy is sufficiently similar to humans. LeDoux applies Tulving’s 3-way scheme linking autonoetic (self-knowing), noetic (knowing) and anoetic (unknowing) forms of consciousness to distinctive forms of memory—episodic, semantic/conceptual and procedural respectively. Procedural memory and the kind of learning it involves provide what he thinks is the best place to start investigating nonhuman mammals for the foundational aspects of anoesis, and he indicates re-representation of subcortical states in medial cortex as the likely mechanism. On LeDoux’s account, nonhuman mammals might have only pre-conscious states because medial cortex activity is not further integrated into the noetic and autonoetic spaces supported by the significant prefrontal expansion in human evolution. Anoetic consciousness (gut feelings and the like) lack specific content, but can shape the experience of human noetic and autonoetic consciousness. His suggested empirical research programme is to probe the common underlying mechanisms of anoetic feelings via the shared anatomy with other mammals. LeDoux insists that careful detailing of the mechanisms underlying a pre-conscious capacity would be a considerable scientific achievement even if the anatomical and behavioural similarities cannot fully undermine the level of scepticism he thinks is appropriate concerning consciousness in nonhumans.

Colin Klein and Andrew Barron (KB) [70] propose a *neurocomputational framework* for investigating phenomenal consciousness in nonhuman animals. They seek a level of abstraction that allows for detailed comparative work that is very broad in scope. They are particularly interested in how insect brains support the kind of computation needed for mobile animals with a capacity for goal-directed behaviour and spatial senses that must be corrected for their own motion. Such organisms must solve the problem of action selection in environments where rewards are highly contingent and variable. This requires them to use sensory inputs, representations of internal state, and stored knowledge of reward value, to output action/expected-value pairs, to select among actions, and to update expected values based on experience. They propose that a ‘phenomenal interface’ provides the common currency in which the values of different actions can be compared. They further propose a transformation-function (π) modelled within the class of nonlinear multi-objective Markov decision processes is mostly likely to be implemented in the insect brain by interactions between the insect mushroom bodies, where valuations are updated, and the central complex (CX) which maintains spatially structured representations of objects. KB ground their approach in the assumption that this is how and where insects evolved a solution to the action selection problem. They set aside scepticism regarding animal consciousness by making an analogy to T.H. Morgan’s initially speculative but highly productive assumption that chromosomes were the basis for heredity. The kind of computational abstraction they seek is not such that any system that moves, keeps track of its location and selects among actions will be phenomenally conscious—it matters how this is done. (Robots and feedforward neural networks need not apply!)

Albert Newen and Carlos Montemayor (NM) [71] lament that most leading theories of consciousness pay little attention to evolution, and claim that an evolutionary perspective will be an important foundation for any successful theory of consciousness. They identify two core types of phenomenal consciousness: basic arousal and general alertness, which is an attention-dependent form of phenomenal consciousness. To this they add a further phenomenon, reflexive (self-)consciousness, which although functionally distinct, is argued to be a form of general alertness but with meta-representational content. Each of these core phenomena plays a distinct functional role in the service of an organism’s survival. The authors then consider some leading theories of consciousness, specifically IIT, higher-order thought and GNWT, and argue that taking an evolutionary perspective concerning these theories and the neuroscientific evidence they cite demonstrates that none is adequate: Because of their cortex-centric focus, the cited theories of consciousness do not adequately account for basic arousal. NM argue that progress on understanding consciousness can be made by paying close attention to the different functional roles of consciousness and to their proposed neural substrates. Successful theories will account for and integrate both.

Last but certainly not least, the review by Jacques Singer and Antonio Damasio (SD) [72] explores the theme of valence as a common currency, already discussed in several previous articles, in explicitly neurocomputational terms. SD explore the distinction between analogue and digital computation in the context of the vertebrate nervous system. The article builds upon Damasio’s many years of arguing that ‘feelings’ (namely, the internal subjective value that experiencers assign to events or internal drives) play a key role in guiding proximately adaptive responses [73–75]. In particular, interoception, and the neural circuits that support it, provide the crucial foundation for subjectivity and the ‘ownership’ feature of consciousness (that my perceptions and actions belong to me). These basic and phylogenetically old features of consciousness are augmented, but not created, by the neocortical systems underlying the ‘modern mind’ of humans and our closest relatives. SD point out that the neural mechanisms underlying interoception have some odd features: they often lack myelin, the fatty sheathing of axons that, in most of our nervous system, acts as insulation from the local environment. SD propose that this openness allows the interoceptive nervous system to ‘commingle’ with the body in a more analogue manner to generate the ‘feeling mind’, in contrast to the mostly digital processing characterizing the neocortical modern mind. SD recognize that this is a provocative new hypothesis. For example, unmyelinated neurons still fire all-or-none action potentials in an arguably digital fashion, and sensors at the initial sensory transduction stage for exteroception (e.g. rods and cones in the retina, or hair cells in the cochlea) emit analogue graded potentials, so their digital/analogue distinction is not a strict dichotomy. They end their review with a call for more research into and thinking about the relatively neglected analogue, feeling aspects of nervous function.

## 5. Conclusion

As these brief summaries illustrate, there is considerable diversity of current opinion about both the functions of consciousness and their phylogenetic extent. In many cases, consideration of the functions of consciousness has opened up novel avenues for potential empirical investigation by generating testable hypotheses. Such research has also broadened the potential phylogenetic scope of consciousness research by moving beyond a simplistic ‘more like humans implies more likely consciousness’ viewpoint, and illustrates how, by testing functional hypotheses, we could empirically evaluate consciousness in birds, insects or cephalopods, despite fundamental differences in their nervous systems. Although we would be the last to argue against the value of neuroscientific investigations of human consciousness, there is a growing recognition that despite considerable research effort, current approaches to the neural correlates of consciousness have led to little consensus (cf. [76]). Furthermore, neuroscientific investigations of human consciousness alone are unable to address some of the fundamental questions about the origins and phylogenetic scope of different forms of consciousness, or the ethical and animal welfare implications such questions raise. We believe that the current collection illustrates the value of asking functional ‘why?’ questions about consciousness across a broad range of species. We hope that it convinces existing consciousness researchers of the value of addressing such functional questions, and inspires researchers in animal cognition and evolutionary/comparative neuroscience to take a new look at consciousness research from a functional perspective.

## Data Availability

This article has no additional data.
